# Evaluation of Disease Activity in Inflammatory Bowel Disease: Diagnostic Tools in the Assessment of Histological Healing

**DOI:** 10.3390/biomedicines11113090

**Published:** 2023-11-18

**Authors:** Alina Ecaterina Jucan, Otilia Gavrilescu, Mihaela Dranga, Iolanda Valentina Popa, Ioana-Ruxandra Mihai, Vasile-Claudiu Mihai, Gabriela Stefanescu, Vasile Liviu Drug, Cristina Cijevschi Prelipcean, Radu-Alexandru Vulpoi, Oana-Bogdana Barboi, Irina Ciortescu, Catalina Mihai

**Affiliations:** 1 Department of Gastroenterology, Saint Spiridon County Hospital, 700111 Iasi, Romania; otilia.gavrilescu@umfiasi.ro (O.G.); gabriela.stefanescu@gmail.com (G.S.); vasidrug@email.com (V.L.D.); cristina.cijevschi.prelipcean@umfiasi.ro (C.C.P.); oany_leo@yahoo.com (O.-B.B.); irinaciortescu@yahoo.com (I.C.); catalina.mihai@umfiasi.ro (C.M.); 2 Faculty of Medicine, “Grigore T. Popa” University of Medicine and Pharmacy, 700115 Iasi, Romania; iolanda-valentina.g.popa@umfiasi.ro (I.V.P.); vulpoi.radu@yahoo.ro (R.-A.V.); 3 Department of Rheumatology and Rehabilitation, Faculty of Medicine, “Grigore T. Popa” University of Medicine and Pharmacy, 700115 Iasi, Romania; ioana-ruxandra_mihai@umfiasi.ro; 4 Department of Radiology, Faculty of Medicine, “Grigore T. Popa” University of Medicine and Pharmacy, 700115 Iasi, Romania; mihaivclaudiu@yahoo.com

**Keywords:** Crohn’s disease, ulcerative colitis, histological healing, endoscopic techniques, surrogate markers, clearance disease

## Abstract

Inflammatory bowel disease (IBD) comprises two types of chronic intestinal disorders: Crohn’s disease and ulcerative colitis. In long-standing ulcerative colitis disease activity, histological persistent inflammation has been linked to an increased risk of relapse, and long-term corticosteroid use, even when endoscopic remission is reached. In Crohn’s disease, the discontinuous nature of lesions and transmural inflammation have limited the standardized histological assessment. The current evidence from research proposes that besides clinical and endoscopic healing, the achievement of histological healing constitutes an endpoint to assess disease activity and remission in IBD patients concerning better long-term disease outcomes. Histological alterations may persist even in the absence of endoscopic lesions. For these reasons, new advanced techniques promise to revolutionize the field of IBD by improving the endoscopic and histologic assessment, disease characterization, and ultimately patient care, with an established role in daily practice for objective assessment of lesions. This review outlines the importance of including microscopic evaluation in IBD, highlighting the clinical benefits of a deep state of disease remission using validated diagnostic methods and scoring systems for daily clinical practice.

## 1. Introduction

Crohn’s disease (CD) and ulcerative colitis (UC) are the two main forms of chronic inflammatory bowel disease (IBD) characterized by an idiopathic inflammatory disorder affecting the gastrointestinal tract. The etiology and pathogenesis of IBD are still unclear. The most common triggers are dysregulated immune response to the commensal gut flora and host genetic and environmental factors [1].

Recent research suggests that in addition to endoscopic healing, the achievement of histological healing (HH) is associated with better long-term outcomes and could represent a potential main goal in managing IBD. The idea of using histological healing as an endpoint to assess disease activity and remission in IBD patients started with the demonstration that treatment with 5-aminosalicylic acid (5-ASA), corticosteroids, immunomodulators, and then biological agents could induce symptomatic relief and also endoscopic and histologic remission [2]. In patients with UC, it has been shown that microscopic activity, even when endoscopic remission is achieved, is an independent risk factor associated with an increased risk of relapses, long-term corticosteroid use, and complications, suggesting the hypothesis that HH could represent a potential therapeutic target [3]. The focus on CD histology has increased recently, but data are still inconsistent and conflicting. The assessment of HH has several limitations due to the discontinuous nature of lesions. However, recent international consensuses have considered that achieving histological remission is an appropriate and realistic goal in both UC and CD clinical trials [4].

Recently, the concept of “disease clearance” has been proposed and described as a profound and complete state of disease remission, comprising symptomatic remission and a “true” mucosal healing state (clinical, endoscopic, and microscopic remission) [5]. However, the Selecting Therapeutic Targets in Inflammatory Bowel Disease (STRIDE II consensus) working group does not yet recommend HH as a treatment target and very few available clinical trials have assessed histological findings among primary outcomes [6], with it quite frequently being specified as an additional exploratory result. Instead, STRIDE-II certifies that the most feasible long-term targets for IBD patients are clinical remission, endoscopic healing, restoration of QoL (quality of life), and absence of disability [7].

This review aims to systematically outline the latest evidence regarding HH in IBD, highlighting the clinical benefits of the microscopic disease activity assessment. We will discuss and provide the reader with the importance of including microscopic evaluation in CD and UC diseases using validated diagnostic methods and scoring systems for daily clinical practice. 

This review uses the terms that define histological endpoints as synonyms and comprise histological healing/remission/response.

## 2. Histological Healing—Current Concept and Clinical Relevance 

We searched the published literature by exploring the PubMed, GoogleScholar, EMBASE, and MEDLINE databases utilizing the following keywords: “inflammatory bowel disease”, “IBD”, “ulcerative colitis”, “Crohn’s disease”, “guidelines”, “histological healing”, “surrogate markers”, “diagnostic tools”, and “disease clearance”, in all possible combinations. We extracted information on diagnosis and management and summarized the current knowledge related to the latest challenges in IBD to promote further research that may improve understanding and help develop clinical practice guidelines for better disease progression and control.

Different authors have defined HH as a mucosa with few architectural abnormalities but normally differentiated epithelial cells and no signs of active inflammation or an increased density of lymphocytes and plasma cells [8]. The European Crohn’s and Colitis Organisation (ECCO) guidelines defined HH as a resolution of crypt architectural distortion and inflammatory infiltrate defined by the absence of intraepithelial neutrophils, erosions, and ulceration as the minimum standard to classify a patient as having achieved histological remission of the bowel mucosa [9]. Therefore, a simplified definition of HH can be described as the microscopic normalization of mucosal biopsies defined by the absence of acute inflammation. Furthermore, in the STRIDE-II committee, HH in UC and transmural healing in CD are considered measures of remission depth and not therapeutic goals [7,10]. However, the STRIDE guidelines’ recommendations focused on daily practice, not clinical trials.

Given the concept that the resolution of intestinal inflammation beyond endoscopic healing could provide clinically relevant advantages or major contributions to patient care, there is increasing interest in assessing histological disease activity. Clinical signs and symptoms combined with endoscopic examination are traditionally used to monitor and assess disease activity status in IBD [11]. HH is achievable in many UC patients and is associated with better disease outcomes than clinical remission and/or endoscopic healing, as revealed by several studies [12]. Evidence indicated that histological remission represents a different target from endoscopic mucosal healing in UC and is associated with lower relapse rates, reduced risk of developing colorectal cancer, and need for surgery or hospitalizations [13,14,15,16,17]. Also, the role of histology as a predictive factor has been explored in several trials in CD [18]. While in UC patients, histological activity is a stronger predictor of clinical relapse, the role of histological assessment in CD has been explored less. From the registered evidence on CD assessment for patients in clinical remission, HH was associated with a lower risk of relapse or hospitalization, improved clinical outcomes, and decreased need for medication escalation or corticosteroid use [19]. As a particularity, in CD a new target and relevant element of healing is transmural healing, assessed by cross-sectional imaging techniques (ultrasound, contrast-enhanced computed tomography, and magnetic resonance enterography) [20]. Table 1 presents a summary of the studies analyzing the association between histological activity disease and clinical relapse.

**Figure 1:**
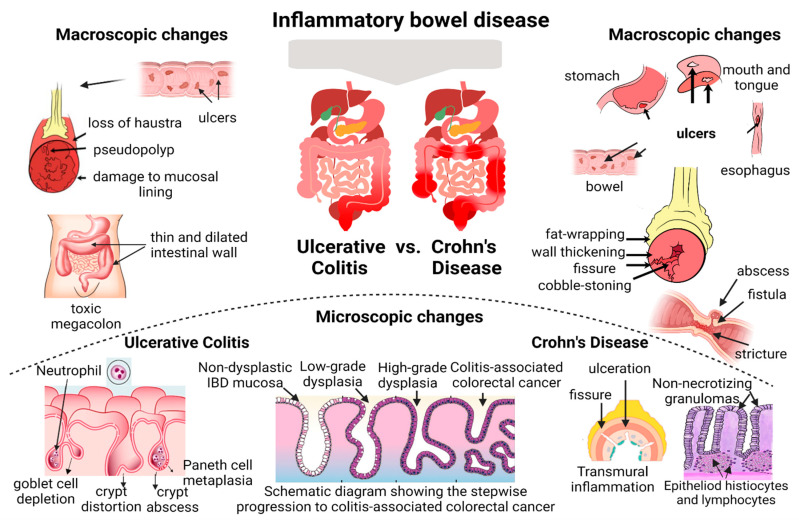
Macroscopic and microscopic changes after a long-standing activity of ulcerative colitis and Crohn’s disease; the histological features that define chronicity are crypt architectural distortion, crypt atrophy, non-necrotizing granulomas, basal plasmacytosis, basally located lymphoid aggregates, and Paneth cell metaplasia; the presence of neutrophils defines inflammatory activity.

In long-standing disease, the persistence of histological inflammation can lead to other complications, such as dysplasia, strictures, fissures and fistulous tracts, perianal manifestations, intermural- or abdominal abscesses, and atrophic mucosal surface. In UC, inflammatory pseudo-polyps may develop as a result of extensive ulcered areas with sparing fields of residual normal mucosa. In CD, ulcerated areas can fuse, and inflammation in the intestines can result in a thickening of the intestinal wall, leading to massed and profound linear ulcers with prominent mucosal edges appearing as patches of cobblestones. Spontaneous bowel perforations to the abdominal cavity are a potentially devastating complication of fissures or fistulas resulting from superimposed ischemia or infection [8]. The risk of colorectal carcinoma in IBD patients is influenced by the chronicity of inflammation during multiple surveillance episodes. A recent meta-analysis conducted by Flores et al. showed that the risk of developing colorectal carcinoma was higher in patients with microscopic activity compared to patients with endoscopic mucosal healing (OR = 2.6 (95% CI, 1.5–4.5; *p* = 0.01)) [29]. Thus, persistent histologic activity is an independent risk factor for developing colorectal carcinoma, and these patients should be considered more frequently at surveillance intervals. Figure 1 reflects the classical features of the long-standing inflammatory activity of UC and CD diseases represented by gross pathology and microscopic changes.

Current drugs and their effect on intestinal inflammation struggle to achieve even endoscopic mucosal healing, which is an earlier target and usually easier to achieve than histological healing. As mentioned above, because patients with histologic persistent inflammation have a higher risk of relapse, the physician might consider optimizing any current medical therapy they are taking. For example, if a patient is taking 5-ASA agents at a low or maintenance dose but has ongoing histologic inflammation, the physician may increase that dose to try to achieve histologic healing [12]. The potency of inducing histologic remission appears to be different depending on the drug; thus, we need more evidence to demonstrate that the resolution of microscopic inflammation as a result of modifying therapy or increasing dose is indeed a superior goal [12]. Therefore, if histologic healing is taken as a treatment target, further data are needed to support and extend these findings. Current guidelines do not yet consider histological healing as a therapeutic target, as more evidence-based studies are needed. Therefore, there are currently no international guidelines in force to assist GI physicians on the specific therapeutic changes imposed by the current state of the histological disease activity.

The value of consensus definitions in studies evaluating HH and establishing relevant cut-offs is needed not only to optimize treatment or a standardized measurement of histological activity but also to change the long-term history of the disease to improve patient outcomes. HH has increasingly become a significant target to achieve; therefore, the importance of histologic remission as a therapeutic aim in IBD continues to evolve.

## 3. Currently Available Diagnostic Tools

Multiple observational studies have demonstrated that histological remission was associated with better clinical outcomes in IBD than endoscopic remission [30]. Numerous methods of classification of histological activity have been proposed, and some are extensively used, with only a few validated and proven to be reproducible. HH occurs later than endoscopic remission, and long-term treatment courses are linked with higher histological remission assessment. 

### 3.1. Histological Healing Scoring Systems—Endoscopic Biopsies

Given the need for standardized quantification of histological activity in clinical trials and routine daily practice, many distinct histological activity scoring systems have been presented during the past decades. The first histopathological changes in colonic and rectal mucosa after hydrocortisone therapy in UC were described in the 1950s by Truelove and Richards [31]. Since then, up to 30 indices have been developed to evaluate histological activity in UC and CD according to the Cochrane Collaboration review [32,33], while only a small number of them have been fully validated. The most extensively used histological scores for UC and CD are summarized in Table 2. Some of the widely used validated histological scores for UC include the Geboes score (original and simplified scores), Robarts histopathology index, and Nancy index.

In 2000, Geboes et al. developed the Geboes score (GS) by using 99 biopsies from UC patients [34]. This score has been widely used in clinical trials and routine practice even though it has never been formally validated. In 2016, a simplified GS (SGS) version was suggested, which was made to reduce its practical complexity [35]. The eosinophilic density in the GS has been reduced to a single scoring variable in indices that were improved later (the Robarts and Nancy indices), taking into consideration its imprecise status as a significant histological trait in IBD [36]. The main strength of the two Geboes scores (GS and SGS) is their ability to assess both acute and chronic histological changes, with precise stratification of both active and inactive UC [37]. Histological remission is defined as GS ≤ 6 (absence of epithelial neutrophils). 

In 2015, the Nancy index (NHI) was developed to assess histological disease activity in UC patients, using 200 biopsies, to provide a scale that was developed by studying 8 features and including only domains that correlated with a global visual evaluation of histopathological severity [38]. This score has been validated for use in routine practice and clinical studies. Histological remission is defined as NHI = 0 (absence of neutrophils in the epithelium, and no erosions or ulcers). According to the ECCO recommendations, NHI is conceptually simple and easy to apply in routine daily practice [39]. The relationship between the NHI and GS was assessed with good responsiveness and correlation between them.

In 2017, Mosli et al. [40] developed the Robarts histopathological index (RHI), mainly derived by comparing the GS and the Riley score using a 100 mm visual analogue score (VAS), capable of evaluating the global grade of inflammation in sample biopsies. The elements of original GS that best anticipated VAS were involved in this new RHI score, including chronic inflammatory infiltrate, lamina propria neutrophils, neutrophils in the epithelium, and erosions or ulcerations [37]. Histological remission is defined in UC as RHI ≤ 3 (sub-scores of lamina propria neutrophils and neutrophils must be equal to 0, with no ulcers or erosions) [36].

The IBD-DCA (Inflammatory Bowel Disease—Distribution, Chronicity, Activity) score is a simple histological activity score, validated by dedicated IBD specialists and applied for both UC and CD providing an accurate treatment response. The unique characteristic of the score is its versatility; hence, it has the potential value of being applied in clinical practice and clinical trials, which signifies that it can be utilized for both UC and CD [41]. For CD, the Global Histology Activity Score (GHAS) score established by D’Haens et al. in 1998 is the only one used on a larger scale to assess early postoperative recurrence after ileocecal resection [42]. Due to its subjectivity, it has not been validated and its role is currently undefined. Validated histologic scores are needed in CD, and further research needs to be collected before concluding. 

**Table 2:** The most widely used histological scoring system in ulcerative colitis (UC) and Crohn’s disease (CD); GS—Geboes score; NHI—Nancy histological index; RHI—Robarts histopathology index; GHAS—Global Histology Activity Score; IBD-DCA—Inflammatory Bowel Disease—Distribution, Chronicity, Activity score.

Author	Score/Index	Year of Publication	Comments	Items
Histological scoring systems in ulcerative colitis				
Geboes et al. [34]; Jauregui-Amezaga, A et al. [35]	Original and Simplified Geboes score	2000; 2017	The main limitation—both scores have not been fully validated Reproducible grading system Histological remission is defined as GS ≤ 6.0 GS ≤ 2.0	Simplified Geboes score Grade 0: No inflammatory activity Grade 1: Basal plasma cells Grade 2A: Eosinophils in lamina propria Grade 2B: Neutrophils in lamina propria Grade 3: Neutrophils in epithelium Grade 4: Epithelial injury (in crypt and surface epithelium)
Gupta et al. [43]	Harpaz score	2007	Partially validated	Grade 0: no cryptitis Grade 1: cryptitis < 50% crypts Grade 2: cryptitis > 50% crypts Grade 3: ulcerations or erosions
Marchal-Bressenot et al. [38]	Nancy histological index (NHI)	2015	Validated and widely used in clinical practice Correlation between the Nancy index and the Geboes index is very good Histological remission defined as NHI = 0	Grade 0: no histological significant disease Grade 1: chronic inflammatory infiltrate with no acute inflammatory infiltrate Grade 2: mildly active disease Grade 3: moderately active disease Grade 4: severely active disease
Mosli et al. [40]	Robarts histopathology index (RHI)	2017	New validated histopathological index Based on Geboes index and modified Riley index Histological remission defined as RHI ≤ 3	Chronic inflammatory infiltrate 0 = no increase; 1 = mild but unequivocal increase; 2 = moderate increase; 3 = marked increase Lamina propria neutrophils 0 = none; 1 = mild but unequivocal increase; 2 = moderate increase; 3 = marked increase. Neutrophils in epithelium 0 = none; 1 = 50% crypts involved. Erosion or ulceration 0 = no erosion, ulceration, or granulation of tissue; 1 = recovering epithelium + adjacent inflammation; 2 = probable erosion focally stripped; 3 = unequivocal erosion; 4 = ulcer or granulation of tissue
Histological scoring systems in Crohn’s disease				
D’Haens et al. [42]	Global Histology Activity (GHAS) Score	1998	Not formally validated The only one used on a larger scale. GHAS score ≥ 10 indicates severe histological activity	Epithelial damage 0 = normal; 1 = focal; 2 = extensive architectural changes 0 = normal; 1 = moderate; 2 = severe mononuclear cells in lamina propria 0 = normal; 1 = moderate increase; 2 = severe increase Neutrophils in lamina propria 0 = normal; 1 = moderate increase; 2 = severe increase Neutrophils in epithelium 1 = surface epithelium; 2 = cryptitis; 3 = crypt abscess Erosion or ulceration 0 = no; 1 = yes Granuloma 0 = no; 1 = yes Number of segmental biopsy specimens affected 1 = < 1/3; 2 = 1/3–2/3; 3 = > 2/3
Histological scoring systems in ulcerative colitis and Crohn’s disease				
Lang-Schwarz et al. [41]	IBD-DCA	2021	Common scoring available for UC and CD Validated by a large group of IBD specialists Provides reliable information on treatment response	Distribution 0 = normal; 1 = < 50% of tissue affected per same biopsy site; 2 = > 50% of tissue affected per same biopsy Chronicity 0 = normal; 1 = crypt distortion and/or mild lymphoplasmacytosis; 2 = marked lymphoplasmacytosis and/or basal plasmacytosis Activity 0 = normal; 1 = two or more neutrophils in lamina propria in one high-power field and/or any presence of intraepithelial neutrophils; 2 = crypt abscesses, erosions, ulcers

According to an official recommendation from the European Society of Pathology (ESP) and the European Crohn’s and Colitis Organisation (ECCO) [44], at least two biopsies from each segment (terminal ileum, right colon, transverse, descending, sigmoid, and rectum) should be taken from every patient with suspected IBD, which must be placed in separate specimen containers [45]. Additional samples should be taken from the endoscopically most affected tissues, especially at the edges of the ulcer. In a recent meta-analysis conducted by Gupta et al. [46] which included 28 studies involving 2806 patients with IBD, the elements that predicted relapse were represented by crypt architectural irregularities, basal plasmacytosis, neutrophilic infiltrations, and mucin depletion.

In summary, according to the ECCO Position Paper: Harmonization of the Approach to Ulcerative Colitis Histopathology, the definition of histological remission in UC was proposed as the absence of intraepithelial neutrophils, erosion, and ulceration as an essential condition. Moreover, different scores are available for the assessment of UC inflammation or activity, and while the GS is extensively used, only the RHI and the NHI have been formally validated [39]. Regarding CD, in agreement with ECCO Position on Harmonization of Crohn’s Disease Mucosal Histopathology, the definition of histological remission as an appropriate histological target should comprise the absence of erosion, ulceration, and neutrophilic inflammation. CD scoring systems differ considerably regarding the epidemiological aspects, the number of segments, clinical presentation, anatomical location, endoscopic and histopathological features, and disease course. The GHAS is the most widely used histological score in CD and registers the activity for each ileo-colonic and rectal site for CD, but this score has not been formally validated [47].

There are several histopathological scoring systems, particularly for UC, which seem to represent a valid tool for including histological remission in routine clinical practice. The histological examination of endoscopic biopsies is an essential element in the IBD diagnosis, evaluation of possible therapeutic effects, and identification of the presence of specific abnormal cells that confirm dysplasia. Optimal management of IBD requires a histological score capable of assessing not only disease activity but also restoration of normal mucosal architecture. Many studies have been performed to develop histological scores of inflammation in IBD, but a small number of them are currently applied, and none are globally used in routine practice. 

### 3.2. New Endoscopic Tools

New insights into personalized and individualized IBD therapy are now available and could be capable of reducing the gap between mucosal and histological healing. Sophisticated advanced endoscopic techniques such as these can provide a deeper ultra-structural characterization of the mucosa with a more comprehensive histological assessment [48].

Confocal laser endomicroscopy (CLE) allows a direct real-time visualization during endoscopy, which provides the opportunity to perform in vivo mucosal microscopic analysis [49]. The principle of CLE is based on illuminating tissue with a low-power laser and then detecting the distribution of fluorescein sodium in tissues, which is the most broadly used fluorescent agent that allows for the visualization of the tissue structure after laser excitation. Different instruments are feasible, but one of the most widely used in clinical practice is probe CLE (pCLE; Cellvizio; Mauna Kea) [50]. pCLE imaging allows both functional and morphological evaluation of the colonic mucosa with high resolution and tissue penetration, as well as the ability to depict microscopic images, such as crypt and microvascular alterations [51]. 

A growing body of studies has evaluated the potential of CLE to assess the histological activity degree and extension of mucosal inflammation in IBD patients. Karstensen et al. [52] and Li et al. [53] outlined an real-time inflammation activity assessment by CLE and showed that all the parameters included (crypt architecture, fluorescein leakage, and vessel architecture) had a good correlation with histopathology (Geboes histology index), differentiating between active and nonactive UC patients during the endoscopic procedure. Specifically in UC in remission, CLE images of crypts have shown small, round, irregularly arranged crypts, compared with the active phase of UC disease, where CLE images have shown severely distorted crypt architecture (87.2% vs. 17.5%) with an irregular surface and increased lamina propria cellularity (89.7% vs. 17.5%; *p* < 0.0001 for all comparisons) [54]. In CD, in comparison with UC, remarkable CLE characteristics showed more significant features of discontinuous inflammation (87.5% vs. 5.1%), focal cryptitis (75.0% vs. 12.8%), and discontinuous crypt architecture (87.5% vs. 10.3%; *p* < 0.0001) [55]. Therefore, CLE appears to be able to differentiate between UC and CD, but its role in evaluating CD may represent limitations compared to UC, due to transmural intestinal changes and CD complications. Moreover, CLE seems to be capable of distinguishing not only between CD and UC, but also appears to be a promising tool for identifying patients at high risk for the development of IBD-associated dysplasia, typical collagenous colitis features, or the diagnosis of Clostridioides difficile colitis [50].

Endocytoscopy (ECS) is an ultra-high magnification endoscopic technique that delivers real-time in vivo microscopic imaging of cells and nuclei at the mucosal surface during ongoing endoscopy. The absorptive agents represented by methylene blue, toluidine blue, or cresyl violet, together with a mucolytic agent (N-acetylcysteine) applied on the mucosa, allow better penetration of the contrast agent, and produce an image close to histology, thus, helping ECS to observe cells and nuclei of mucosal surfaces [56]. 

Several activity scores have been developed over time, and the first endocytoscopy score (ECSS) was developed by Bessho et al. to grade disease activity accurately by assessing the shape and the distance between crypts and the visibility of microvessels [57], with good predictive value for the histopathological activity of UC. In a recent study involving a total of 64 UC patients in clinical and endoscopic remission (Mayo endoscopic score of 0), Nakazato et al. showed that the ECS score was strongly correlated with histological activity. The ECS score revealed high accuracy in detecting histological remission (sensitivity of 0.77, specificity of 0.97, and diagnostic accuracy of 0.86) and can be used to assess HH in UC patients without the need for biopsy samples [58]. Another endocytoscopic evaluation of microscopic disease activity in UC based on the newly developed scoring system ELECT (ErLangen Endocytoscopy in ColiTis) score demonstrated a strong correlation with valid histopathology scores (Robarts histopathology index, r = 0.70; Nancy histologic index, r = 0.73). The overall ELECT score was calculated as the sum of all subcategories from five parameters (crypt shape, crypt distance, vascular architecture, inflammatory cell infiltrate, and crypt abscess), ranging from 0 to 6. Moreover, ECS was superior to white-light endoscopy for the grading of histologic activity, with good performance measures in terms of sensitivity, specificity, and accuracy of 88%, 95.2%, and 91.3%, respectively [59]. 

Recent breakthroughs in artificial intelligence (AI) have sparked increasing interest in implementing CAD systems as a new method to improve the quality of IBD endoscopy. Maeda et al. [60], in a retrospective study involving the data of 187 patients with UC, developed and evaluated a computer-aided diagnosis (CAD) system that uses artificial intelligence (AI), based on an endocytoscopy system, to predict the persistent histologic activity and long-term clinical prognoses. The CAD system provided performance measures regarding sensitivity (74%), specificity (97%), and accuracy (91%). Despite the encouraging results, more studies are needed to validate the role of using ECS combined with AI to achieve considerable proficiency before its application in routine practice. Furthermore, in a prospective study enrolling 29 UC patients, ultra-high magnification endocytoscopy scores strongly correlated with histological scores of RHI ≤ 3 or NHI ≤ 1. Also, the study examined the potential molecular pathways and soluble markers/expressed genes that could predict histological remission defined by ultra-high magnification with histology scores [61]. ECS can probably evaluate a relatively wide area of the colonic mucosa with an accurate evaluation of histological inflammation, but the high costs and dedicated training to achieve good proficiency could be a limitation for its use in the daily practice of IBD management. 

Virtual electronic chromoendoscopy (VCE) is extensively available in most endoscopic units and leads to a better assessment of vascular patterns and mucosal surface features, which helps differentiate between persistent inflammation versus quiescent disease, a distinction that is increasingly recognized as a key therapeutic purpose in IBD patients [62]. For example, in UC, various new scores, such as the Paddington International Virtual Chromoendoscopy Score (PICaSSO) [63], have been developed by using VCE. A large multicenter international study has developed a strong correlation between PICaSSO (the only validated and reproduced score that all endoscopic platforms can use) and five histological scores (RHI, NHI, Villanacci Simple Score, Geboes Score, and Extent and ECAP (Extent, Chronicity, Activity, and Plus score)), significantly superior to correlation coefficients of MES and UCEIS with histology scores. PICaSSO < 3 predicted good long-term outcomes at 6 and 12 months with an HR of 0.19 and 0.22, respectively [64]. Moreover, combined endoscopic–histologic remission measured with VCE PICaSSO was increasingly explored compared with endoscopic remission alone as an ultimate goal in UC for predicting clinical outcomes at 12 months [65].

A meta-analysis conducted by Nardone et al. [66] could accurately distinguish between endoscopic and histologic disease activity scores in UC and found that both VCE and WLE are strongly correlated with histology, with more accuracy for VCE in predicting histological remission (risk ratio: 1.13, 95% confidence interval (CI): 1.07–1.19, *p* < 0.001). More recently, a study conducted by Iaccuci et al. [67] developed the first computer model based on am artificial intelligence (AI) system to evaluate endoscopic remission or activity and predict the histological remission and risk of acute flare in UC from white-light endoscopy (WLE) and VCE videos. The study involved 1.090 endoscopic videos from 283 patients to develop a convolutional neural network (CNN) to detect inflammation/healing on VCE using the PICaSSO prospective multicenter international study [64]. The AI system detected endoscopic remission using the Ulcerative Colitis Endoscopic Index of Severity (UCEIS ≤ 1) in WLE videos with a sensitivity and specificity of 72% and 87%, respectively, and an area under the receiver operating characteristic curve (AUROC) of 0.85. When employing VCE videos (endoscopic remission defined as PICaSSO ≤ 3) sensitivity improved to 79%, specificity to 95%, and AUROC to 0.94. Moreover, the prediction of histological remission (defined as RHI ≤ 3 and no neutrophils in lamina propria) was similar between WLE and VCE videos (sensitivity, specificity, and accuracy of 67%, 86%, and 81% using WLE, and 73%, 86%, and 83%, respectively, using VCE videos) [67]. 

Concerning CD, the current approach for scoring disease activity can predict the achievement of endoscopic remission [68], but regarding the assessment of histological remission, there is a lack of research literature, and further studies on this topic are required. To summarize, VCE is accurate in predicting histological remission and can be used as a surveillance colonoscopy technique, and PICaSSO score can evaluate inflammation accurately, making endoscopy closer to histology. 

In conclusion, the development of new advanced techniques promises to revolutionize IBD endoscopy and histology by improving disease characterization and, ultimately, patient care. Moreover, in addition to the histological assessment of IBD, the early detection of dysplasia is one of the major challenges in IBD endoscopy, and an AI system would be of great help in detecting precursor dysplastic lesions or early colorectal cancer [69].

## 4. Surrogate Markers for Histological Healing

In recent years, the relationship between endoscopy and surrogate markers of inflammation has gained attention and research interest, but there is little evidence regarding their involvement in predicting histological remission. Various promising non-invasive biomarkers that appear to correlate well with histological disease activity are currently being investigated and are showing promising results [70]. According to recent research, the ideal biomarker should be simple, sensitive, non-invasive, disease-specific, and reproducible, and should correlate with the severity of damage and satisfy three different fields: patient compliance, reliability for the disease, and kinetic stability [71]. Endoscopy is the gold standard for evaluating IBD patients, but due to its high costs and disease burden on patients, numerous research attempts in the IBD field have been directed towards finding accessible and cost-effective biomarkers that can be used to quite easily perform tests without affecting patients’ quality of life.

### 4.1. Common Biomarkers Predicting Histological Healing

Previous studies have demonstrated that common biomarkers, such as CRP (C-reactive protein), are not disease-specific [70]. CRP is an important monitoring serum biomarker and can be sufficiently assessed during the active phase of IBD, and it is suitable for assessing disease activity and therapy efficacy by repeated measurements in clinical practice. Elevated CRP levels could differentiate active mucosal inflammation from inactive IBD. At the same time, the assessment of HH or disease remission when CRP is negative is contested, mainly because of its lower accuracy in patients with lower activity [72]. Moreover, no correlation between histological remission and CRP as a surrogate marker for histological activity has been established in the current literature. 

The fecal immunochemical test (FIT) is another marker that has been reported in a few studies to be applicable in the assessment of mucosal healing in UC [73,74]; however, its accuracy to assess for HH has not yet been validated. The correlation between FIT and two extensively used histological scores (the Nancy index ≤ 1 and the Geboes score < 2.0) was moderate and accurate in predicting HH in UC. The area under curve (AUC) of FIT was comparable to that of fecal calprotectin (FC) for HH (*p* = 0.767–0.960) and was comparable to colonoscopy (*p* = 0.384–0.673). FIT < 50 ng/mL predicted HH with a sensitivity, specificity, and positive predictive value of 73–75%, 67%, and 78–80%, respectively. Moreover, the study showed that combining FIT with FC led to a higher specificity (90%), and over 85% of patients with FIT < 50 ng/mL and FC < 50 μg/g achieved HH [75]. Thus, FIT appears to be a reliable non-invasive marker for HH in UC patients, but larger longitudinal studies are needed to validate the cut-off value of FIT. 

FC has been revealed as a feasible indirect measure of inflammation. It appears to be the most popular and well-studied non-invasive biomarker in diagnosing and monitoring IBD patients, but its suboptimal testing properties do not allow it to be used as an alternative to endoscopy. A growing body of research identified the optimal FC cut-off level to predict histological remission in UC. The correlation between FC and histological activity found that an optimal FC cut-off of ≤60 mg/g accurately predicted deeper remission with good performance measures (AUC = 0.91, MES = 0, Nancy index ≤ 1, specificity and sensitivity of 90% and 83%, respectively) [76]. Recently, a systematic review enrolling 12 studies and 1168 patients stated the association between FC levels and histological activity in UC patients showing a clear correlation between FC levels and histology. FC cut-off levels varied across studies and were able to discriminate histological remission from histological activity, varying from 40.5 to 250 μg/g. Hence, it has been stated that identifying FCP cut-off levels requires larger prospective trials using validated histological indices [77]. Walsh et al. [78] demonstrated a strong correlation between FC and endoscopic activity [r = 0.741] or histopathology [r = 0.876]. FC thresholds for detecting histological disease activity are anticipated to be more stringent than those for endoscopic activity, and, indeed, the FC cut-off for histological inflammation was ≥72 μg/g for Nancy ≥ 2 [AUC 0.824]. Another study conducted by Hart et al. [79] observed that an FC level ≥135 µg/g predicted histologic activity. Larger prospective trials using validated histologic scores are needed to find a worldwide accepted FC cut-off level to distinguish between patients with persistent active histologic disease and those in histological remission.

Another study involving fecal biomarkers FC and lactoferrin (FL) showed a close correlation with histological activity and could differentiate between patients with histological disease activity from those in histological remission [80]. FC levels were significantly increased among patients with evidence of active histological disease (NI ≥ 2; median 69.72 [IQR 20.07–254.38]), compared to those without (NI ≤ 1; median 12.35 [IQR 3.89–32.16]); z = −6.60, *p* < 0.001). By comparison, FL concentrations were considerably increased among patients with active histological disease (NI ≥ 2; median 18.59 [IQR 6.06–44.42]), compared to those without (NI ≤ 1; median 3.14 [IQR 0.75–11.05]); z = −5.70, *p* < 0.001). Optimized cut-offs for FC (≥34.29) and FL (≥5.85 μg/g) enhanced the accuracy compared to the manufacturer’s cutoffs (FC: 69.9% vs. 65.9%; FL: 71.7% vs. 69.0%) [80]. Therefore, patients with elevated FC and FL are likely to have active histological inflammation.

The performance of Leucine-rich alpha-2 glycoprotein (LRG) on mucosal healing was evaluated in a trial involving 166 UC patients and 56 CD patients [81]. LRG value was compared with those of CRP, FIT, and FCP and was not superior to fecal markers. Instead, in CD patients, the performance of LRG was equivalent to that of CRP and FCP. A significant correlation with histological activity was found for each biomarker (Spearman’s rank correlation coefficient: LRG: 0.21, *p* = 0.0091, CRP: 0.17, *p* = 0.035, FIT: 0.51, *p* < 0.0001). Therefore, fecal markers might be preferred for IBD patients with low disease activity compared to LRG [81]. No longitudinal data or studies in larger populations were available to assess the microscopic activity and establish the cutoff value of LRG on HH; thus, further studies are required.

To summarize, CRP value could be considered during the active phase, while FC and FL seem to be advantageous in the active histological disease; LRG was not superior to fecal markers, while FIT can predict HH in UC, but larger trials and longitudinal data are needed to validate its cut-off value. However, more evidence is needed to explore their potential as predictive biomarkers.

### 4.2. Novel Biomarkers Predicting Histological Healing

The discovery of an IBD biomarker, Cytokine Oncostatin M (OSM), a member of the IL-6 cytokine family has attracted much interest in recent years. OSM and its receptors (OSMR) are positively correlated with histological severity in IBD patients, and they are highly expressed in both inflamed tissue and blood. West et al. demonstrated that OSM is the most consistently expressed cytokine in inflamed intestinal tissues of IBD patients and could be a potential biomarker and therapeutic target for IBD, especially for anti-TNF-resistant patients [82]. Increased colonic OSM levels have been associated with poor prognosis and with primary non-response to biological treatment. In an analysis of 30 patients with IBD, serum OSM and FC levels were evaluated concerning clinical and endoscopic scores in IBD patients, and the value of OSM as a predictive marker of treatment response at baseline and one year of follow-up treatment was also examined. The ROC curve for OSM in the prediction of treatment response revealed that the best cut-off value between groups regarding the OSM level was <103.50 pg/mL, with a sensitivity of 40% and specificity of 75%. [83]. OSM appears to be a promising biomarker in the tissue and serum of patients with IBD, involved in the pathogenesis, diagnosis, and follow-up of IBD patients, with a notable relationship between its levels and degree of severity. Still, its accuracy and potential value in predicting HH in IBD require further investigation.

MicroRNAs (miRNAs) are small, non-coding RNA molecules that can regulate gene expression at the posttranscriptional level. These markers can be easily detected in whole blood, serum, stool, and intestinal tissues, and have the potential to be used as disease markers in assessing the severity and treatment response of IBD patients [71]. Recently, more than 800 fecal miRNAs were measured in stool tests of IBD patients and compared to controls. The results suggest that CD and UC patients in the active phase of the disease have distinct fecal miRNA profiles compared to healthy controls, particularly including higher levels of miRNA-223 and miRNA-1246 [84]. Larger studies are required to assess the full potential and the significance of miRNA in the assessment of treatment response, and endoscopic and histological remission in IBD patients. Nevertheless, differential expression of specific miRNAs has been involved in disease etiopathogenesis, and the inflammatory status and the biopsy site must be considered when elucidating the role of miRNAs [85].

In the literature, there are also attempts to find composite markers. A recent study by Bertani et al. showed that in UC patients, scores based on the activity of serum cytokines, at baseline and over the first 6 weeks of treatment with vedolizumab, may assess the treatment response. If considering the IBD complexity and heterogenicity, it is obvious that a single biomarker cannot predict the disease course and that the combination of several specific biomarkers is certainly an area that needs to be expanded, as there is a lack of data on this specific issue [86,87]. Findings on composite scores of IBD activity have begun to emerge in recent years, but in terms of histological activity, it would be useful to have a quantifiable measure to provide existing inflammation that indicates the state of tissue damage.

Several promising biomarkers for IBD have been identified, but there is a lack of standardization of histological procedures, definitions, and scoring systems. At present, no biomarker is accurate enough to replace endoscopy. Even though the relationship between endoscopy and surrogate markers of inflammation has gained increasing interest in recent years, there is little data available on their role in predicting histological remission. Although more assessments should focus on specific biomarkers, comparative studies, and more clinical trials are also needed to establish the full potential of new biomarkers to impact clinical care.

## 5. Future Directions—Beyond Histological Healing in IBD

Many observational studies have suggested that, in real life, symptomatic, clinical, endoscopic, and histological remission have low achievement rates, for both CD and UC [88]. In recent decades, new biological treatments admitted to clinical practice have had promising results with histological remission in IBD patients. Also, with the development of the molecular biology field and recent progress in understanding immunologic pathways in IBD, new pharmacologic therapies have been revealed [89]. Therefore, disease clearance could be regarded as a potential new treatment target in IBD, reducing future complications and flares with better disease progression and control. Moreover, one of the most recent challenges of modern gastroenterology in IBD, molecular healing, will be a future direction for predicting disease outcomes [90].

A few years ago, the treatment goals for IBD were focused on improving clinical symptoms and quality of life, but the targets have evolved into more desirous ones by targeting the natural history of the disease. Tapping into disease clearance components can block long-term disease progression and prevent complications that lead to surgery, disability, and the risk of neoplasia in IBD patients. Therefore, clinical trial objectives have recently been developed to achieve these goals.

Danese et al. [5] have recently proposed the concept of disease clearance defined as a state of complete and profound remission (endoscopic, histological healing, evolving towards molecular healing) with the restoration of distinct molecular targets involved in the pathogenesis of the disease as the ultimate target in the treatment of UC patients. The natural history of patients with UC could be modified by achieving several goals involved and defined as normalization of the stool frequency, with the absence of rectal bleeding, endoscopic remission (MES = 0), a decrease in fecal calprotectin value (<150 mg/g), histological remission/inactivity (GS ≤ 2 or NI = 0), and normalization profile in the molecular study of the biopsies [91]. During a multicenter retrospective cohort study with extended follow-up (24 months) of 109 UC patients with clearance disease (clinical remission, Mayo score = 0, Nancy index = 0), a significantly reduced risk of hospitalization (5.5% vs. 23.1%; *p* < 0.001) and surgery (1.8% vs. 10.9%; *p* = 0.003) was observed compared with the control group [92]. The first head-to-head study, the VARSITY trial, compared vedolizumab with adalimumab in UC patients’ treatment and demonstrated superior performance for vedolizumab in achieving disease clearance at week 52 compared to adalimumab (29.2% vs. 16.3%). The disease clearance definition in the VARSITY trial included an MES ≤ 1 and a RHI < 5 [93,94]. Moreover, the ongoing VERDICT (Determination of the Optimal Treatment Target in Ulcerative Colitis) trial NCT04259138 is the first multicenter, prospective, randomized, controlled trial in moderately to severely active UC to assess whether patients’ treatment response after using a corticosteroid-free symptomatic, endoscopic, and histologic remission is superior to corticosteroid-free symptomatic remission alone. The primary endpoint is the time to a UC-related complication within a maximum of 80 weeks of follow-up after reaching the target. This is a disease modification trial; thus, it aims to assess whether the natural history course of the disease can be modified. Early results of the clinical trial consider the feasibility of reaching each treatment target, in particular histologic remission, and are consistent with expected values [95].

Regarding CD, the definition of disease clearance is less defined and may comprise transmural healing as best appreciated by cross-sectional imaging, such as ultrasonography, CTE, and MRE, as mentioned above. Transmural inflammation in CD is one of the defining characteristics of the disease, the endoscopic examination cannot always be complete and does not evaluate transmural changes, and discontinuous inflammation introduces sampling error for histological assessment [96]. However, as mentioned above, the concept “treat to target” in the new consensus STRIDE considers HH in UC and transmural healing in CD as measures of remission depth [7].

The best predictor of disease clearance may still be early therapy, and the history of the disease could increase the possibility of achieving disease clearance. For example, it is easier to achieve disease clearance when the disease is less severe and has less endoscopic or histological activity. When several episodes of acute flare with deep ulcerations and severe endoscopic activity occur during the disease course, disease clearance is more difficult to achieve [97]. Also, it is important to step up therapy when a patient is in clinical remission but has not achieved endoscopic and histologic remission, to increase the efficacy of drugs by finding biomarkers of response to a drug, and/or to combine drugs to increase the likelihood of healing and of achieving disease clearance [98].

In addition, the road to IBD precision medicine is still challenging. In-depth research activity in recent years in newer in-vivo histology techniques provides the basis for a more comprehensive analysis of the underlying molecular pathways. Molecular imaging devices, such as fluorescence endoscopy and near-infrared fluorescence endoscopy can be combined with VCE, thus, enabling highly individualized and specific description of intestinal mucosal inflammation in the future [99]. Bioinformatics tools that outline the integrative personal profiling for precision medicine, including immune profiling, genetics, transcriptomics, microbiota analysis and imaging, and molecular endoscopy, will soon revolutionize IBD management, improving the accuracy of diagnosis, disease progression monitoring, and targeted therapies [100]. 

In this area of research, there are many gaps, including validation and standardization of the concept of disease clearance, possibly beyond histological and transmural healing towards molecular healing, and the influence of distinct treatments and therapeutic strategies on the evolution and prognosis of the disease. It is important in further prospective trials to demonstrate that follow-up of disease clearance correlates with better long-term patient outcomes than clinical, endoscopic, and histological remission and has a positive risk–benefit ratio.

## 6. Conclusions

In the past few years, numerous innovative and mandatory steps have been taken in the diagnosis, management, and treatment of IBD, with the histological examination of endoscopic biopsies considering the concept of HH as a therapeutic target. IBD patients with persistent inflammation are at significantly increased risk of developing more frequent relapses, cumulative damage, and disability, leading to elevated risk of hospitalization, colorectal cancer, and surgical procedures. Histological healing is a possible goal different from endoscopic mucosal healing, associated with better disease outcomes and reduced disease-related complications compared to clinical remission and/or endoscopic healing. Furthermore, recent advances in endoscopic technology have been developed, and many are beginning to be applied in clinical routine, providing accurate and precise descriptions of the histology of vascular architecture (including sparse vessels, crypt architecture, and cellular infiltration). Also, a validated and standardized histological score could lead to a more precise definition of microscopic activity for both clinical practice and clinical trials. 

Therefore, HH is increasingly being considered an important new goal to achieve, and, consequently, further histological evaluation in IBD needs to validate the role of histopathology in clinical trials and practice, mainly for patients in clinical and endoscopically apparent remission.

## Figures and Tables

**Table 1:** The association between histological activity and the risk of clinical relapse. A *p*-value < 0.05 is considered statistically significant; CR—clinical relapse; OR—odds ratio; CI—confidence interval; NQHA—normal or quiescent histological activity; CHA—chronic histological activity; AHA—acute histological activity; NHI—Nancy histopathological index; HR—hazard ratio.

Study	Type of Study	Disease	N Patients	Endoscopic Activity	Histological Index	Outcome
Park et al. [21]	Systematic review and meta-analysis	UC	1360 patients	Endoscopic remission	Truelove and Richards index; Riley index; Geboes score. Histological remission- present in 964 patients (71%).	52% relative risk reduction in relapse/exacerbation for UC patients with histologic remission compared to histologic activity.
Narang et al. [22]	Prospective observational study	UC	76 patients in clinical remission for at least 6 months. 46 patients with endoscopic remission included (Mayo score ≤ 1; 46/76, 60.5%), 1 year of follow-up.	Endoscopic remission	Geboes score; Histological remission in 67.3% (31/46) of patients, while 32.7% (15/46) with histologically active disease.	87.1% (27/31) of patients with histological remission remained asymptomatic, while 12.9% (4/31) had relapsed. Among histologically active patients, 46.6% (7/15) sustained clinical remission, while 53.3% (8/15) had relapsed. (87.1% vs. 46.6%, *p* = 0.006).
Ozaki et al. [23]	Prospective study	UC	194 patients, 20 months of follow-up.	Endoscopic remission	NHI was significantly higher in MES 1 than in MES 0 [1.11 ± 0.09 vs. 0.41 ± 0.07, *p* < 0.0001].	67 patients relapsed during the follow-up period; risk of relapse (HR- 2.18 [1.16–5.82]; *p* = 0.03).
Bryant et al. [24]	Prospective study	UC	91 patients, 6 years of follow-up.	Endoscopic remission	24% of patients had persistent inflammation.	Histological remission predicted lower rates of corticosteroid use and acute severe colitis requiring hospitalization during follow-up (HR 0.42 (0.2 to 0.9), *p* = 0.02; HR 0.21 (0.1 to 0.7), *p* = 0.02, respectively).
Bessissow et al. [25]	Cohort study	UC	75 patients, 12 months of follow-up	Endoscopic remission	Geboes score ≥3.1 in 40% and basal plasmacytosis in 21% of patients.	The presence of basal plasmacytosis, predictive of CR; OR = 5.13 (95% CI: 1.32–19.99), *p* = 0.019.
Calafat et al. [26]	Retrospective observational study	UC	113 patients underwent dysplasia surveillance colonoscopy between January 2005 and October 2015; follow-up of 12 months was included. The median time of follow-up—2.5 years.	Endoscopic remission	62 patients (57%) presented NQHA, 33 (30%) presented CHA, and 22 (20%) presented AHA. Basal plasmacytosis- present in 9 patients (8%), six of them in association with AHA (5%).	9 patients (8%) relapsed within the first year of follow-up and 37 patients (33%) relapsed during the whole follow-up period. The presence of AHA is a risk factor for clinical relapse.
Christensen et al. [27]	Retrospective study	CD	101 patients, follow-up for a median of 21 months.	63% of patients with endoscopic remission.	55% of patients achieved histologic remission.	CR occurred in 42% (n = 42) of patients Histologic healing was associated with a decreased risk of CR (HR- 2.05; 95% CI, 1.07–3.94; *p* = 0.031).
Brennan et al. [28]	Retrospective cohort study	CD	62 patients, follow-up for at least 6 months. A total of 103 patients with CD underwent elective colonoscopies during clinical remission.	55 patients (53%) in endoscopic healing, 48 patients (47%) with active disease.	A semiqualitative score (0 to 3) was assigned for the histologic characteristics in each of the biopsy samples.	At 12 months, the rate of relapse was 25.5% in patients with histologic activity, compared with only 2.4% of patients without histologic activity at baseline. The presence of histological activity was associated with higher flare rates (*p* < 0.05).
